# Visualization of the intrarenal distribution of capillary blood flow

**DOI:** 10.14814/phy2.14065

**Published:** 2019-04-22

**Authors:** Letao Fan, Shaoxun Wang, Xiaochen He, Ezekiel Gonzalez‐Fernandez, Claude Lechene, Fan Fan, Richard J. Roman

**Affiliations:** ^1^ Department of Pharmacology and Toxicology University of Mississippi Medical Center Jackson Mississippi; ^2^ Center of Nanoimaging Brigham and Women's Hospital Cambridge Massachusetts

**Keywords:** Blood vessels, capillaries, kidney, renal blood flow

## Abstract

This study describes a modified technique to fill the renal vasculature with a silicon rubber (Microfil) compound and obtain morphologic information about the intrarenal distribution of capillary blood flow under a variety of conditions. Kidneys and cremaster muscles of rats were perfused in vivo with Microfil using a perfusion pressure equal to the animal's mean arterial pressure at body temperature. Microfil did not alter arteriolar diameter or the pattern of flow in the microcirculation of the cremaster muscle. The modified protocol reproducibly filled the renal vasculature, including; glomerular, peritubular, and vasa recta capillaries. We compared the filling of the renal circulation in control rats with that seen in animals subjected to maneuvers reported to alter the intrarenal distribution of blood flow. Infusion of angiotensin II, hypotension, volume expansion, and mannitol‐ or furosemide‐induced diuresis redistributed flow between renal cortical and medullary capillaries. The advantage of the current technique is that it provides anatomical information regarding the number, diameter, and branching patterns of capillaries in the postglomerular circulation critical in determining the intrarenal distribution of cortical and medullary blood flow.

## Introduction

The complex organization of the renal vasculature has been well described (Ljungqvist and Lagergren 1962; Moffat and Fourman 1963; Plakke and Pfeiffer 1964). Previous investigators (Speller and Moffat 1977) concluded that the regional perfusion of the kidney is divided into four structurally distinct vascular zones: the cortex, the outer stripe of the medulla, the inner stripe of the outer medulla (frizzled zone), and the inner medulla. Blood flowing into the cortical peritubular capillary network derives from the efferent arterioles of cortical glomeruli. Perfusion of the other zones of the kidney arises from blood exiting juxtamedullary glomeruli via efferent arterioles. Alterations in regional blood flow are due to changes in the diameter and flow in a constant number of perfused capillaries or alterations in the number of perfused vessels within a region of interest. Thus, morphologic information about the pattern of perfusion within the kidney, the number of perfused vessels, their diameters, and branching patterns is critical to understand the mechanisms underlying changes in capillary blood flow within the kidney. Moreover, changes in blood flow between superficial versus deep cortical nephrons along with redistribution of flow between cortical peritubular, and medullary vasa recta capillaries have long been suspected to influence sodium excretion and urine concentrating ability. However, the mechanisms involved are still poorly understood, in part because of a lack of technique to visualize the pattern of postglomerular capillary perfusion in vivo.

Many techniques have been employed to measure changes in regional blood flow in the kidney (Knox et al. 1984). Aukland et al. (1964, 1968) implanted electrodes to measure relative changes in blood flow by hydrogen clearance in the superficial versus deep cortex of dogs. Radiolabeled microspheres have also been employed to assess the distribution of blood flow in cortical versus juxtamedullary glomeruli following volume expansion and administration of vasoactive factors (Birtch et al. 1967; Mcnay and Abe 1970). Haywood et al. (1981) and Mattson et al. (1993) employed laser‐Doppler flowmetry to measure cortical and medullary blood flow in rats. These approaches allow for the assessment of changes in blood flow at defined points within the renal circulation but provide little information about the redistribution of flow among capillary beds within the postglomerular network.

Roman et al. (1988), Pallone et al. (2011), and Yamamoto et al. (2002) employed videomicroscopy to observe the flux of red blood cells (RBCs) in individual vasa recta capillaries. These studies revealed that the velocity of RBCs in vasa recta as well as the number of perfused vessels are altered in response to elevations in perfusion pressure, volume expansion, angiotensin, vasopressin, and other vasoactive agents. However, the diameters of these vessels were unchanged, so the changes in flow were most likely secondary to alterations in relative vascular resistance between branches in the upstream capillary network.

Two‐photon microscopy (Dunn et al. 2002; Gyarmati et al. 2018) and a charge‐coupled video microscope with a pencil‐lens (Yamamoto et al. 2002) have been used to image blood flow in individual glomerular and peritubular capillaries. However, these techniques are limited to measurements in individual capillaries within 500 *μ*m of the surface of the renal cortex. They have not been used to measure the flux of RBCs in the renal medulla or changes in the intrarenal distribution of blood flow. Moreover, while videomicroscopy and two‐photon microscopy are excellent tools to study the vascular diameter and the velocity of RBC in single arterioles and capillaries, the field of view and the depth of the focus are too narrow to provide much information about the distribution of blood flow between capillaries within postglomerular vascular networks.

Garcia‐Sanz et al. (1998) developed a technique for filling the renal vasculature in situ with Microfil after perfusion fixation and then imaging preglomerular vessels using three‐dimensional microcomputed tomography (Micro‐CT). However, even with advanced imaging techniques and long scan times, the poor contrast between filled and unfilled tissue, the overlapping nature of the vessels within the vascular network, and the current insufficient resolution of Micro‐CT all limit imaging of afferent arterioles, glomerular capillaries, or peritubular capillary network (Garcia‐Sanz et al. 1998; Hlushchuk et al. 2017; Ngo et al. 2017). As such, Micro‐CT studies of the renal vasculature using Microfil remain focused on the study of angiogenesis, rarefaction, and remodeling of larger preglomerular arteries > 30 *μ*m in diameter (Hlushchuk et al. 2017; Ngo et al. 2017). More recently, several groups applied dynamic multidetector computed tomography (CT) to measure relative perfusion of the cortex and medulla of man and larger animals (Perrien et al. 2016; Hlushchuk et al. 2017; Epah et al. 2018). Others have taken advantage of blood‐oxygen‐level‐dependent magnetic resonance imaging (BOLD‐MRI) to assess changes in tissue oxygenation as an index of changes in regional blood flow (Prasad 2006; Gloviczki et al. 2011, 2013). These methods provide important information regarding changes in blood flow in the renal cortex versus outer medulla, but lack the resolution to visualize individual vessels or the number of perfused capillaries within the region of interest. Overall, the results of previous studies have provided strong evidence that redistribution of blood flow between cortical and vasa recta capillaries is involved in pressure natriuresis acute kidney injury, and the responses to atrial natriuretic factor, volume expansion, as well as the pathophysiology of many diseases of the kidney (Roman and Zou 1993; Pallone et al. 2011). However, very little information is available about the changes in the pattern of perfusion within the postglomerular capillary networks under these conditions.

Microfil has been widely used to study renal tubular–vascular relationships (Barger and Herd 1973; Beeuwkes and Bonventre 1975), and as a contrast agent to study changes in the structure of the preglomerular arteries and arterioles, especially in large animals by Micro‐CT (Chade 2013). However, it is far more difficult to fill and image the postglomerular circulation, especially in rodents, due to the higher vascular resistances of the afferent arteriole and glomerular, peritubular, and vasa recta capillaries (Nordsletten et al. 2006; Chade 2013; Perrien et al. 2016; Ngo et al. 2017). The present study describes modifications of the original method of Sobin et al. (1962) to prepare renal vascular casts, which allows for more complete and reproducible filling of the renal vasculature of rats with Microfil, including the postglomerular capillary circulation. We verified that our modified technique does not alter vascular reactivity by comparing the diameters of vessels in the cremaster muscle measured in vivo using videomicroscopy versus those obtained after filling the vessels with Microfil. We further evaluated whether this technique could provide information about the regional perfusion of the kidney by comparing the pattern of perfusion of cortical peritubular versus medullary vasa recta capillaries in control rats with that seen in animals subjected to maneuvers reported to alter the intrarenal distribution of blood flow. The results indicate that infusion of angiotensin II, hypotension, volume expansion, and mannitol‐ or furosemide‐induced diuresis redistribute blood flow between cortical and medullary capillaries. These findings are consistent with the changes in blood flow in deep versus superficial glomeruli reported in previous studies using microspheres or laser‐Doppler measurements of cortical versus medullary blood flow (Birtch et al. 1967; Roman et al. 1988; Mattson et al. 1993; Roman and Zou 1993). The main advantage of the present technique is that it provides anatomical information regarding the number of perfused capillaries, their diameters, and branching patterns needed to determine mechanisms involved in redistributing blood flow within the postglomerular capillary circulation.

## Material and Methods

### General

Experiments were performed on 80 female Wistar rats weighing between 190 and 390 g. All animals were anesthetized with an intraperitoneal (*i.p*.) injection of thiobutabarbital sodium (100 mg/kg, Sigma‐Aldrich, St. Louis, MO) and were placed on a heated surgical board to maintain the body temperature at 36–37°C. A tracheotomy was performed, and the right carotid artery was cannulated with PE‐50 polyethylene tubing. Blood pressure was continuously recorded using a transducer (Harvard Apparatus, Millis, MA). The right jugular vein was cannulated to allow intravenous (*i.v*.) infusions, and a catheter was placed in the left ureter for urine collection.

A large diameter polyethylene cannula (PE‐280) that was tapered to a tip of 0.3” O.D. over a 1‐cm length was introduced into the abdominal aorta via the left iliac artery. The cannula was filled with a heparinized (10 mU/mL) 0.9% sodium chloride solution. A ligature was placed around the abdominal aorta between the right renal and the superior mesenteric arteries. After surgery and a 30‐min equilibration period, the rats then received one of the nine treatments described below. Prior to filling of the kidneys with Microfil, arterial pressure was directly recorded using a transducer attached to a catheter in the carotid artery, and a timed urine sample was collected to determine urine flow, sodium excretion, and osmolality.

#### Group 1 – Euvolemic control rats

Eleven normally hydrated euvolemic rats were infused with 0.9% sodium chloride solution at a rate of 2.0 mL/h throughout the experiment.

#### Group 2 – Antidiuretic, water‐deprived rats

Food and water were removed from five rats for 18 h prior to the experiment. These animals received an *i.v*. infusion of 0.9% sodium chloride solution at a rate of 1 mL/h throughout the experiment.

#### Group 3 – Volume expansion with isotonic sodium chloride solution

Nine normal rats received an *i.v*. infusion of isotonic 0.9% sodium chloride solution at a rate of 6 mL/h throughout the experiment.

#### Group 4 – Hypertonic 2.0% sodium chloride diuresis

Twelve rats received an *i.v*. infusion of 2.0% sodium chloride solution at a rate of 6 mL/h throughout the experiment.

#### Group 5– Osmotic diuresis

Six rats received an infusion of a 10% mannitol solution *i.v*. at a rate of 6 mL/h throughout the experiment.

#### Group 6 – Furosemide diuresis

These animals were infused with 0.9% sodium chloride solution at a rate of 2.0 mL/h throughout the experiment. Fifteen rats were given a bolus *i.v*. injection of furosemide (25 mg/kg) followed by a continuous infusion at a rate of 0.3 mg/min for 15 min prior to the injection of Microfil.

#### Group 7 – Hypotensive hemorrhage

Arterial pressure was lowered to approximately 50 mmHg in five rats by withdrawing 3–5 mL of arterial blood prior to the infusion of Microfil into the kidney.

#### Group 8 – Angiotensin II infusion

These animals received an infusion of 0.9% sodium chloride solution at a rate of 2 mL/h throughout the experiment. Five minutes prior to the Microfil injection, angiotensin II was infused *i.v*. at a rate of 150 ng/kg/min in six rats. Seven additional rats were given a 30 ng/kg/min nonpressor infusion of angiotensin II.

### Microfil injection procedure

The Microfil perfusate was diluted to a viscosity similar to that of blood by taking 5 mL of MV‐122 yellow silicon rubber compound (Flow Tech Inc., Carver, MA) and mixing it 1:3 with 15 mL of the supplied diluent, instead of the recommended 1:1 dilution. The solution was heated to 37°C and degassed under vacuum for 30 min. Five minutes before perfusion of the kidneys, the animals were given 0.1 mL of sodium heparin (1000 U/mL) by *i.v*. injection. Then, the cannula in the femoral artery was advanced in the abdominal aorta to 3 cm below the left renal artery. Dibutyltin dilaurate catalyst (0.7 mL, 3% v/v) was added to the Microfil perfusate, and the perfusion catheter was attached to a 20‐mL syringe containing the injection media. The Microfil was perfused by occluding the ligature on the abdominal aorta above the kidneys, and applying a pressure to the syringe equal to the control arterial pressure measured in each animal using a CO_2_ gas tank and a two‐stage regulator. Perfusion pressure in the system was monitored using a pressure gauge attached to a side port of the infusion catheter. The left renal vein was cut to allow the injection media to flow freely from the kidneys. The perfusion rate varied between 4 and 6 mL/min. The perfusion was continued for 3 min until 12 mL of media was injected. The kidneys were left undisturbed in the animals for 15 min to allow for complete polymerization of the Microfil. The kidneys were then removed, rinsed with a 0.9% sodium chloride solution, decapsulated, and placed in a 25% aqueous solution of ethanol. After 24 h, the kidneys were sectioned along their longitudinal axis and successively dehydrated for 24 h in 50, 75, and 100% ethanol solutions. Thereafter, the kidneys were placed in methyl salicylate for 1 week to clear the tissue. After clearing, the kidneys were sliced longitudinally into 1‐mm sections. These sections were imaged against a black background in a methyl salicylate bath at 20× using a stereomicroscope equipped with an imaging device.

### Effects of Microfil on vascular tone

Experiments were performed to confirm that the filling of the vasculature with Microfil does not alter vascular diameter. Four rats were prepared, as described earlier for in vivo perfusion of Microfil into the abdominal aorta. In addition, the cremaster muscle of these animals was prepared for intravital videomicroscopy imaging of the microcirculation, as previously described by Lombard et al. (1984). The internal diameters of fourth‐order arterioles of the cremaster muscle were measured in vivo during a control period. The presence of active tone in these vessels studied was confirmed by observing transient dilation in response to topical application of adenosine (10^−4^ mol/L). After the control measurements were obtained, the cremaster muscle was perfused with Microfil, as described above. Fifteen minutes were allowed for polymerization of the Microfil, and the diameters of the arterioles were remeasured in situ. Differences in vessel diameters before and after filling with Microfil were assessed using a paired *t*‐test. A *P* value < 0.05 was considered statistically significant.

## Results

### Influence of Microfil on the vascular diameter

The mean diameter of the fourth‐order arterioles in the cremaster muscle averaged 15.1 ± 1.1 *μ*m (*n* = 12 vessels, 4 rats) when perfused with blood during the control period. After perfusion of Microfil, the diameters of the vascular casts measured in the animal without clearing the tissue were not significantly altered and averaged 15.8 ± 1.3 *μ*m. Examination of the videorecording of the experiments indicated that arteriolar diameters and the pattern of perfusion in the cremaster muscle remained unchanged after filling of the vessels with Microfil. Topical addition of adenosine (10^−4^ mol/L) caused arteriolar dilation and allowed Microfil to fill more vessels.

### Filling patterns of the renal vasculature with Microfil

**Figure 1:**
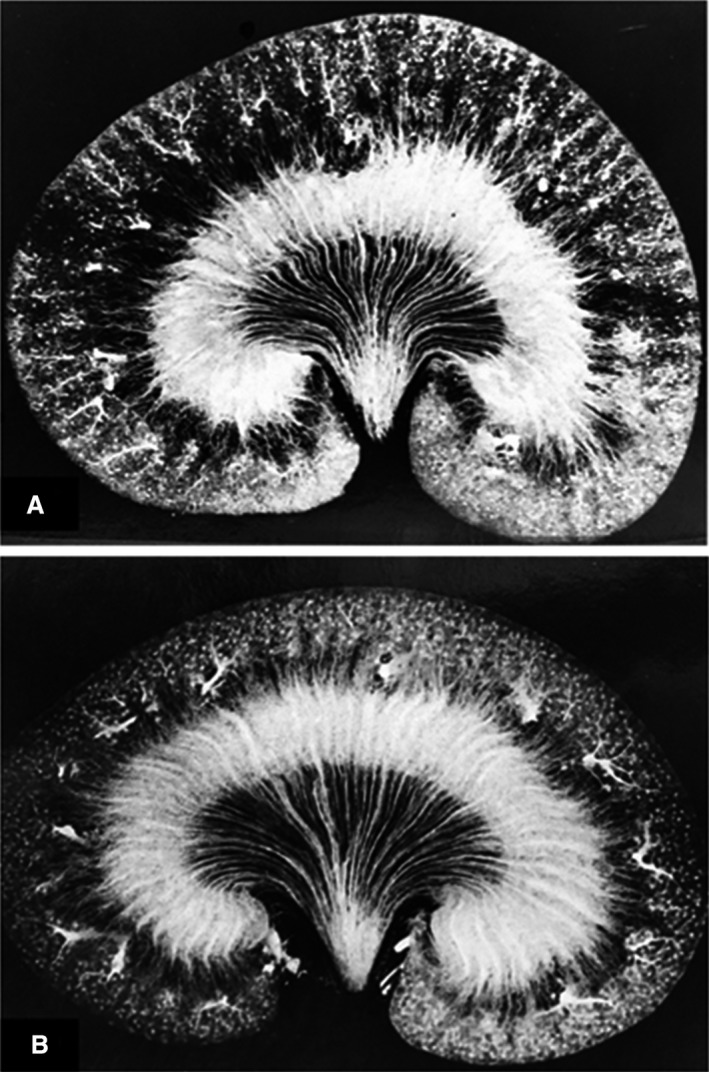
(A) Microfil injected kidneys obtained from a normally hydrated rat and (B) a rat after 16 h of water deprivation.

There was minimal animal‐to‐animal variation in the pattern of vascular filling within experimental groups. However, it was readily apparent that the filling of postglomerular microcirculation was reproducibly altered between groups of rats studied under different conditions. The filling pattern of the renal vasculature in the normally hydrated euvolemic rats excreting urine with an osmolality of 1000–1600 mOsm/kg·H_2_O is depicted in Figure 1A. A kidney representative of dehydrated rats (group 2) excreting highly concentrated urine with an osmolality greater than 2000 mOsm/kg·H_2_O is also presented in Figure 1B. The pattern of filling with Microfil was similar in euvolemic rats to that seen in animals subjected to 24 h of dehydration. All areas of the renal microvasculature were well filled. The four zones of vascular distribution: cortex (superficial and deep), outer medulla, and inner medulla were clearly delineated.

**Figure 2:**
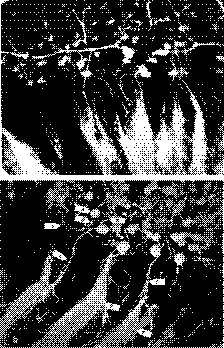
Representative images comparing the filling of the renal cortical and juxtamedullary circulation using the original method for preparing Microfil described by Sobin et al. (1962) and Garcia‐Sanz et al. (1998) versus our modified method are presented in Figure 2. Preglomerular arcuate and interlobular arteries were filled using the normal procedure, but the filling of glomerular and peritubular capillaries in the renal cortex was incomplete (Fig. 2a). Some afferent arterioles perfusing juxtamedullary glomeruli and vasa recta capillaries were also filled. In contrast, both the pre‐ and postglomerular circulation including; cortical peritubular capillaries and medullary vasa recta were well filled using the modified procedure (Fig. 2b). Arrows point to the glomerular capillaries (A), afferent (B) and efferent arterioles (C), peritubular (D), and vasa recta capillaries (E).

A comparison of the filling of the renal circulation using the original procedure for preparing Microfil described by Sobin et al. (1962) and Garcia‐Sanz et al. (1998) versus our modified method is presented in Figure 2. Consistent with previous reports, we could fill interlobular arteries using the normal procedure, but the filling of glomerular and peritubular capillaries in the renal cortex was incomplete (Fig. 2a). Some afferent arterioles perfusing juxtamedullary glomeruli and vasa recta capillaries were filled. This is consistent with previous reports that the diameter of afferent arterioles is larger, and exposed to a higher pressure, in the deep versus superficial nephrons, and the existence of shunts from preglomerular arteries to some vasa recta vascular bundles. In contrast, glomerular capillaries (A), afferent (B) and efferent arterioles (C), peritubular (D), and vasa recta capillaries (E) were all well filled using the modified procedure (Fig. 2b). Vascular diameters, lengths, the number of perfused capillaries, and the branching pattern within the capillary network could readily be discerned.

**Figure 3:**
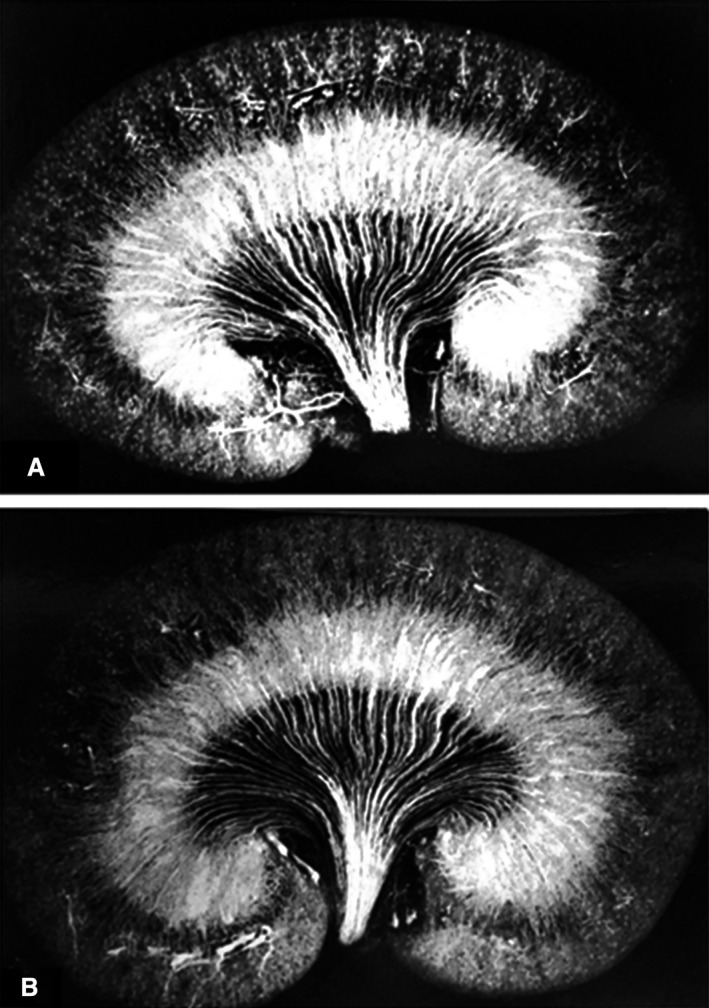
Microfil injected kidneys obtained from (A) a volume‐expanded rat infused with isotonic 0.9% sodium chloride solution or (B) a hypertonic 2.0% sodium chloride solution.

**Figure 4:**
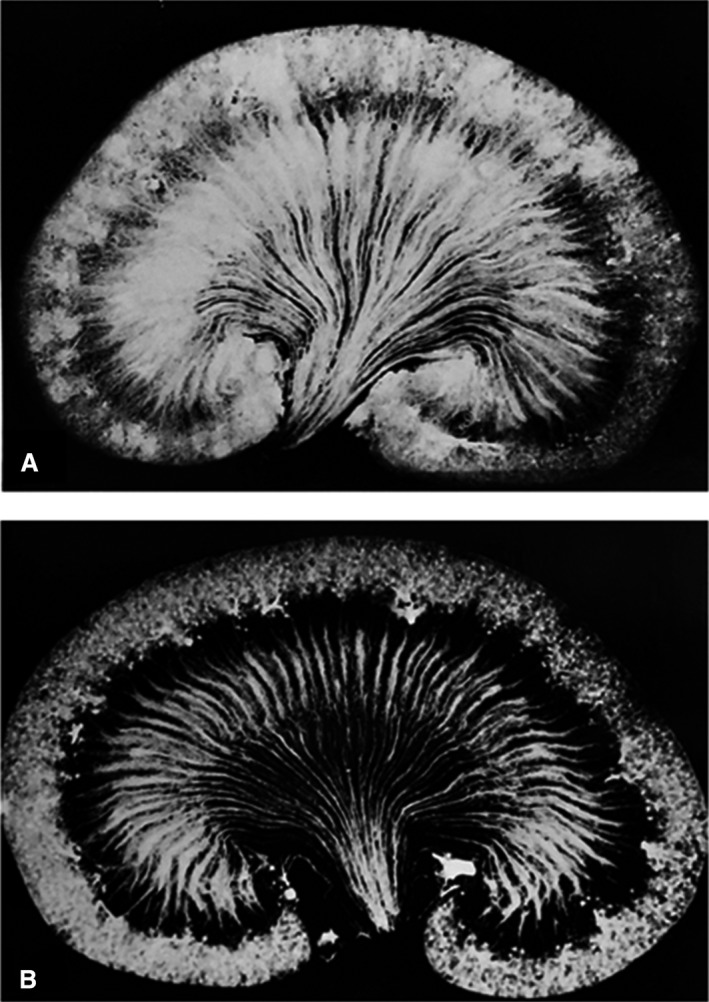
Microfil injected kidneys obtained from a rat undergoing a (A) mannitol or (B) furosemide diuresis.

Volume expansion with an isotonic (Fig. 3A) or hypertonic sodium chloride solution (Fig. 3B) did not alter the pattern of filling in the kidney relative to that seen in euvolemic animals. The appearance of the renal vascular casts was markedly altered in rats undergoing mannitol or furosemide diuresis, relative to that seen in the normally hydrated animals. As depicted in Figure 4A, the filling of the cortical and medullary vasculature increased in animals undergoing a mannitol diuresis. In animals given furosemide (Fig. 4B), the capillaries between the vascular bundles in the inner zone of the outer medulla (fizzled zone) were not filled with Microfil, even though the rest of the kidney was well perfused. There was also a reduction in the filling of vessels in the subcortical zone in the furosemide‐treated rats due to the reduced number of descending vasa recta filled with Microfil.

**Figure 5:**
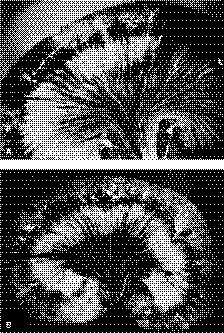
Microfil injected kidneys obtained from (A) a rat subjected to hypotensive hemorrhage or (B) infused intravenously (*i.v*.) with a nonpressor dose of angiotensin II. Similar results were seen in rats infused with a pressor (150 ng/kg/min) dose of angiotensin II.

The results obtained in rats subjected to hypotensive hemorrhage or rats infused with angiotensin II are presented in Figure 5. The filling of the outer cortex in rats subjected to hypotensive hemorrhage was poor and spotty (Fig. 5A), while the filling of the medullary regions of the kidney was better preserved. In contrast, the inner medulla and some regions of the outer medulla of the kidney were poorly filled in rats receiving pressor or nonpressor doses of angiotensin II (Fig. 5B). There were also regions of poor filling in some areas of the renal cortex (left side), but overall, the filling of the renal cortex was better preserved than that seen in the outer medulla.

## Discussion

The present study describes a modification of the Microfil injection technique of Sobin et al. (1962), which allows for more complete and reproducible filling of the kidney, especially the postglomerular capillary circulation of rats. The original Microfil vascular injection procedure has been widely used to prepare vascular casts and as a contrast media for Micro‐CT studies of remodeling of preglomerular renal arteries in a variety of animals (Sobin et al. 1962; Barger and Herd 1973; Grayson et al. 1974; Braun 1976; Garcia‐Sanz et al. 1998; Chade 2013). In general, the use of this technique in the kidney of rodents has been limited, since the filling of the renal vasculature is often incomplete, even when the kidneys were injected after being flushed with vasodilators and perfusion fixed. Several investigators have reported that it was not possible to fill the postglomerular capillary circulation, especially vasa recta and peritubular capillaries of smaller species such as rats and mice (Ngo et al. 2017; Nordsletten et al. 2006 and Perrien et al. 2016).

The modifications employed in the present study were: (1) to use a silicon rubber compound with a lower viscosity than that employed in previous studies; (2) to increase the dilution of the Microfil perfusate from 1:1 to 1:3 to reduce its viscosity to that of blood; (3) to degas the injection media; and (4) to inject the compound at 37°C using a perfusion pressure equal to the mean systemic blood pressure of the animal. With these modifications, we found that the kidneys of rats were well perfused at a rate comparable to the level of renal blood flow. Degassing the elastomer and warming of the compound were essential to prevent constriction of the renal microvasculature. The results of the present study indicate that it is possible to fill all elements of the renal circulation, including the afferent and efferent arterioles, glomerular capillaries, and postglomerular peritubular and vasa recta capillaries (Fig. 2B). One can even discern changes in the perfusion of the very fine capillaries interconnecting ascending and descending vasa recta capillaries in the frizzled zone of the outer medulla.

Further evidence that the diameters of arterioles are not altered during the filling procedure was obtained from the comparison of vascular diameters in the cremaster muscle before and after filling the vessels with Microfil. These results indicated that infusion of Microfil in vivo does not alter the diameter of even small fourth‐order arterioles. Moreover, examination of the videorecordings suggested that it had no effect on the pattern of flow within the cremaster bed. However, it is possible that this may not be true in renal arterioles. If the renal arteries become hypoxic and dilate during the filling procedure, the measured diameter may more closely reflect the passive luminal diameters seen in vessels perfused with Ca‐free media rather than the basal diameters with tone. However, the diameters of isolated perfused afferent arterioles only increase by 10–15% in Ca‐free media, and the effects of hypoxia on the capillaries are even less. At very least, the filling of the renal circulation with Microfil can still provide useful information regarding structural changes and vascular remodeling between strains and experimental conditions and to establish baseline passive diameters of the vessels for modeling studies. Overall, the present findings indicate that in vivo vascular perfusion of Microfil is useful for obtaining morphologic information about the length, diameter, branching pattern of vessels in microcirculatory beds and the number of perfused vessels per unit area of tissue. Changes in vascular volumes per gram of tissue can also be obtained by digesting the tissue in nitric acid and weighing the vascular cast.

One caveat is that clearing the tissue after perfusion with alcohol and methyl salicylate has been reported to shrink the Microfil cast and reduce vascular diameters by about 20% (Møller et al. 1994; Ngo et al. 2017). Shrinkage, however, is not a major concern when exploring the pattern and degree of filling under various conditions, as in the present study. However, if accurate measurements of vascular diameters are required, the tissue can be sectioned and diameters measured without clearing (Ngo et al. 2017). Alternatively, the tissue can be cleared in glycerol that does not shrink the cast (Møller et al. 1994), or larger preglomerular vessels can be studied without clearing the tissue using Micro‐CT (Nordsletten et al. 2006; Chade 2013; Perrien et al. 2016; Ngo et al. 2017).

We performed studies in groups of animals that were infused with different drugs or previously reported to alter medullary blood flow. We found that the pattern of vascular filling was remarkably similar between rats studied under a given experimental condition, but it was altered in groups of rats subjected to different experimental conditions. As can be seen by comparing the images in Figure 1 to those in Figures 4 and 5, the filling of the postglomerular capillary circulations in rats given mannitol, furosemide, or angiotensin II was markedly distinct from that seen in the control rats. On the other hand, isotonic or hypertonic volume expansion had little effect on the pattern of vascular filling. These studies suggest that the distribution of flow in the postglomerular capillary beds is likely dependent on the relative vascular resistances in different capillary beds at the time of the injection. This idea is also consistent with the very rapid nature of the Microfil filling procedure. The capillaries go from being perfused with blood to being filled with an inert, hydrophobic compound within seconds. The compound also polymerizes very rapidly and the vascular cast does not appear to expand, even if the vessels dilate with time after the vasculature has been filled.

We utilized the Microfil injection technique to study the intrarenal distribution of blood flow under a variety of physiological conditions. Examination of the pattern of vascular filling in rats undergoing mannitol diuresis indicated that blood flow through all zones of the kidneys increased. This conclusion is consistent with the previous results of Pilkington et al. (1965) and Goldberg and Lilienfield (1963) in dogs treated with mannitol.

Horster and Thurau (1968) first proposed that shifts in the distribution of blood flow from salt‐retaining juxtamedullary to salt‐losing superficial cortical nephrons could play an essential role in the renal adaptation to a high salt diet and the rapid excretion of a salt load. While high salt diets and acute volume expansion have been repeatedly reported to be associated with increased blood flow at the whole kidney level (Barger and Herd 1973), their influence on the intrarenal distribution of blood flow remains unclear. The results of radioactive gas washout studies by Hollenberg et al. (1970) suggested that high salt diets favor increased blood flow in the superficial layers of the renal cortex. Previous microsphere studies, however, indicated that inner cortical blood flow increased more than flow in the superficial cortex (Stein et al. 1972; Migdal et al. 1975). In the present study, the pattern of filling of the renal vasculature was similar in hydrated and dehydrated rats as well as in rats that were volume expanded with an isotonic or hypertonic solution of sodium chloride. These findings suggest that the distribution of blood flow in the postglomerular capillary circulation is not altered when the sodium or water balance of the animal is changed. Instead, they indicate that blood flow to the different regions in the kidney likely increases in parallel.

The filling pattern of renal vasculatures in furosemide‐treated rats suggests that its diuretic effect is associated with constriction of the descending vasa recta vessels supplying the outer medullary capillary plexus. This observation supports previous findings using radioactive gas washout techniques that outer medullary blood flow is reduced in dogs given furosemide (Birtch et al. 1967). It is also consistent with the report of Stein et al. (1972) indicating that blood flow to the inner cortex measured using microspheres decreased in dogs given furosemide. Since furosemide blocks reabsorption of sodium and chloride in the thick ascending limb of Henle, which lies between the outer medullary vasa recta vascular bundles and is perfused by the outer medullary capillary plexus, it is possible that the furosemide‐induced vasoconstriction is secondary to decreased metabolic demand by the tubules in this region. This conclusion is also supported by increases in tissue oxygenation detected by BOLD‐MRI (Gomez et al. 2009). The rise in the BOLD signal is thought to be due to a decrease in tissue oxygen utilization secondary to inhibition of the Na‐K‐2Cl transporter by furosemide.

The results of the hypotensive hemorrhage studies indicated that medullary blood flow was better preserved than in cortical regions when the kidney was subjected to a low perfusion pressure. This finding supports the original observations of Trueta (1948) showing that the time necessary for the dye to reach the papilla was reduced far less than that of the cortex following a reduction in renal perfusion pressure. This conclusion is also consistent with the results of a variety of other studies using radioactive microspheres (Mcnay and Abe 1970; Stein et al. 1972).

The influence of angiotensin II on the intrarenal distribution of blood flow remains unsettled. A few studies using microspheres (Rector et al. 1972) or radioactive gas washout techniques (Aukland 1968; Barger and Herd 1973) suggested that angiotensin II lowers the inner and outer cortical blood flow without altering the intrarenal distribution of blood flow. On the other hand, Carriere and Friborg (1969) reported that angiotensin II infusion reduces outer medullary blood flow more than cortical blood flow using an autoradiographic method. More recent findings by Lu et al. (1993), Mattson et al. (1993), Spitalewitz et al. (1982), and Faubert et al. (1982) suggested that manipulation of the renin–angiotensin system causes alterations in papillary blood flow, without changing deep cortical blood flow. It supports the view that angiotensin II may selectively constrict postglomerular vessels supplying the vasa recta circulation. The present results, depicted in Figure 5 that angiotensin II produced a more pronounced reduction in the filling of capillaries in the inner medulla than those in the cortex, provide further support for a role of angiotensin II in the regulation of blood flow in the vasa recta circulation. This result is also consistent with the studies of Pallone and Huang (2002) indicating that angiotensin decreases the diameter of vasa recta capillaries by constricting pericytes.

In summary, an improved technique for in vivo filling of the renal microvasculature with Microfil has been presented. Microfil injected in this way did not alter the arteriolar diameters or the pattern of flow in the microcirculation of the cremaster muscle of rats. Using this approach, it was possible to reproducibly fill and image vessels throughout the entire postglomerular circulation, including the afferent and efferent arterioles, glomerular, peritubular, and vasa recta capillaries down to diameters < 10 *μ*m. The filling pattern of the renal vasculature with Microfil in the superficial and deep cortex, outer medulla, and inner medulla was reproducibly altered under different physiological conditions, which indicates that the distribution of Microfil reflects the intrarenal distribution of blood flow in the kidney. Moreover, the pattern of vascular filling induced by infusion of angiotensin II, furosemide, mannitol, and hypotensive hemorrhage in the present study corresponds well with the relative changes in cortical and medullary blood flow reported in previous studies using a variety of approaches. The advantage of the current strategy is that it provides valuable anatomical information regarding the number of perfused capillaries, their diameters, and branching patterns critical to determining the mechanisms involved in altering the intrarenal redistribution of blood flow in postglomerular capillaries, and to develop computational models linking changes in medullary blood flow with sodium and water excretion and urine concentration and dilution.

## Conflict of Interest

None declared.
